# MiR-19a as a prognostic indicator for cancer patients: a meta-analysis

**DOI:** 10.1042/BSR20182370

**Published:** 2019-05-14

**Authors:** Yizhong Peng, Donghua Huang, Kaige Ma, Xiangyu Deng, Zengwu Shao

**Affiliations:** Department of Orthopaedics, Union Hospital, Tongji Medical College, Huazhong University of Science and Technology, Wuhan 430022, China

**Keywords:** cancer, meta-analysis, miR-19a, prognosis

## Abstract

MiR-19a was aberrantly expressed in various types of cancers and was observed to be potentially associated with the prognosis of cancer patients. The present analysis aims to elucidate its precise predictive value in various human malignancies. Online electronic searches of PubMed, Web of Science (WOS), Embase in English and VIP, Wanfang, SinoMed, and the China National Knowledge Infrastructure (CNKI) in Chinese up to September 8, 2018 were conducted. As a result, in overall analysis, a significant association was identified between miR-19a levels and OS (HRs = 2.31, CI: 1.11–4.83). The relation of miR-19a expression to OS was further recognized by fixed model within the studies of sample size less than 150 (HRs = 1.68, CI: 1.35–2.08), NOS scores greater than or equal to 8 (HRs = 1.53, CI: 1.13–2.06) or less than 8 (HRs = 1.89, CI: 1.58–2.27), specimen derived from tumor (HRs = 1.73, CI: 1.42–2.12) or blood (HRs = 1.87, CI: 1.46–2.40) and the patients of osteosarcoma (HRs = 7.17, CI: 5.04–10.21). Sensitivity analyses revealed no significant results. The association between miR-19a expression level and DFS was also found to be significant (HRs = 2.03, CI: 1.13–3.66). Correlations between miR-19a levels and clinicopathological features were examined and revealed that lymph node metastasis was significantly associated with miR-19a expression levels (OR = 0.565, CI: 0.346–0.921). Summarily, the over expression of miR-19a was an underlying risk of poor prognosis in many human malignancies, especially in osteosarcoma. Moreover, elevated miR-19a expression was linked to the potential of lymph node metastasis.

## Background

Cancer which is a multi-factorial disease has been one of the most common causes of death world mortality [1]. In 2012, the world estimated incidence and death rate are 14.09 million and 8.2 million, respectively [2]. Based on the development trend, the new cases will reach 22.2 million in 2030 [3]. Cancer itself and its medical treatment have been a heavy economic burden for both society and family. However, due to the complex interactions between molecular abnormalities and cancer pathogenesis, there lacks of efficient and specific markers in cancer diagnosis and prognosis nowadays.

MicroRNAs (miRNAs), which are small-length, non-coding, and single-stranded RNAs, can act as endogenous RNA interference [4]. By negatively regulating the expression of their target genes at the post-transcriptional level, miRNAs can control a wide variety of physiological functions including cellular proliferation, differentiation, and apoptosis [5]. Many miRNAs have been reported to be aberrantly expressed in carcinomas and can operate either tumor-promoting or tumor-suppressing functions [6,7]. The diagnostic and prognostic characteristics of many miRNAs have been explored in several cancer types recently [8,9]. Therefore, regulated miRNAs have promoted considerable interest in applying them as prognostic markers and therapeutic targets for human cancers [10].

MiR-19a, a member of highly conserved miR-17-92 cluster sited on chromosome 13q31.3, has been observed to play an oncogenic function in various cancer types [11–20]. The expression of miR-19a has been reported to be up-regulated in leukemia, breast cancer, esophageal squamous cell carcinoma, cervical cancer, colorectal cancer, and lung cancer, indicating its linkage with tumor initiation [21–25], apoptosis suppression [26], cell growth [27,28], and lymph metastasis [24]. However, there were a small number of studies exploring an antioncogenic role of miR-19a by inhibiting cells migration and invasion [29], immunoregulation [30], thus associated with a good prognosis of malignancies [31,32]. Hence, we comprehensively collected published cohort studies and performed a meta-analysis to further investigate the role of miR-19a in the prognosis of human cancers.

## Methods

### Literature search strategy

Online electronic searches of PubMed, Web of Science (WOS), Embase in English and VIP, Wanfang, SinoMed and the China National Knowledge Infrastructure (CNKI) in Chinese up to September 8, 2018 were conducted using the following keywords: ‘microRNA-19a’ or ‘microrna-19a’ or ‘miRNA-19a’ or ‘miR-19a’ and ‘tumor’ or ‘cancer’ or ‘carcinoma’ or ‘neoplasm’ or ‘malignancies’ and ‘prognosis’ or ‘survival’. We also retrieved articles manually from other sources, such as the reference lists of review articles.

### Eligibility criteria

Studies from the initial searches that satisfy the criteria below are thought to be eligible. (1) associations of miR-19a expression levels with prognosis or clinicopathological features were reported; (2) describing the outcomes, which include overall survival (OS), recurrence-free survival (RFS), progression-free survival (PFS), or disease-free survival (DFS); (3) patients were dichotomized into two groups in terms of high and low expression levels of miR-19a; (4) the miR-19a expression levels in the cancer patients were measured by some specific methods, such as qRT-PCR (real-time polymerase chain reaction), microarray, etc. Exclusion criteria for the articles included: (1) studies without reporting data with relevant values; (2) duplicated publications or overlapping data; (3) letters, reviews, expert opinions, and case reports; and (4) articles published in languages other than English or Chinese.

### Data extraction

Two independent authors (Yizhong Peng and Donghua Huang) reviewed and extracted the data from each eligible study. The following information was collected: author; year of publication; country; tumor type and clinical stage; number of patients enrolled; the type of specimen; detection methods of miR-19a expression levels; follow-up period; and cut-off values; survival analysis methodology; source of hazard ratio (HR) and HR for OS, RFS, PFS, and DFS as well as their 95% confidence intervals (95%CIs) and quality of study. Sources of HR were directly determined by univariate and multivariate analyses in most of studies, whereas others only provided Kaplan–Meier curves. For the latter studies, using Engauge Digitizer version 9.8, we obtain necessary data from Kaplan–Meier curves and then input the extracted survival rates at specific points of time into the spread sheet developed by Tierney et al. to get the HR and their 95%CI [33].

### Quality assessment

To determine the quality of all the enrolled studies, the Newcastle–Ottawa Scale (NOS) method [34,35] was conducted by three independent authors. Any inconsistency was solved by a senior author. The NOS scores ranged from 0 to 9, and high quality was regarded if a study has an NOS score more than 6.

### Statistical analysis

We accessed the impact of miR-19a expression levels on clinical prognosis by testing the pooled hazard ratios (HRs) and corresponding 95%CIs. An observed HRs > 1 indicated poorer prognosis in patients with increased miR-19a expression. The results were considered statistically significant if *P*<0.05 or the 95%CI did not overlap with 1. The *χ*^2^-based *Q* test and *I*^2^ statistic were conducted to examine heterogeneity across studies. Significant heterogeneity was defined as *P*<0.1 or *I*^2^ > 50%. The fixed-effects model and the random-effects model were both applied to analyze the pooled outcomes. To examine possible sources of between-study heterogeneity, subgroup analyses leveled by sample size (<150 vs. ≥150), NOS scores (<8 vs. ≥8) and tumor category (osteosarcoma vs. non-small cell lung cancer vs. others types) was performed. Moreover, meta regression was further carried out to evaluate the contribution of these potential covariates to the variation of pooled HR. To test if the outcomes were sensitive to restrictions on the data included, sensitivity analyses were conducted by sequential omission of each study. To assess whether the enrolled studies are a representative sample of the available evidence or not, potential publication biases were estimated by Begg’s test and Egger’s test. The odds ratios (ORs) and corresponding 95%CIs were also calculated to test the correlation between miR-19a expression and clinicopathological characteristics. All *P*-values were two tailed, and *P*<0.05 was thought to be statistically significant. Statistical analyses and graphical representations were managed by Stata software version 14.0 (Stata Corporation, College Station, TX, U.S.A.).

## Results

### Characteristics of the enrolled studies

**Figure 1:**
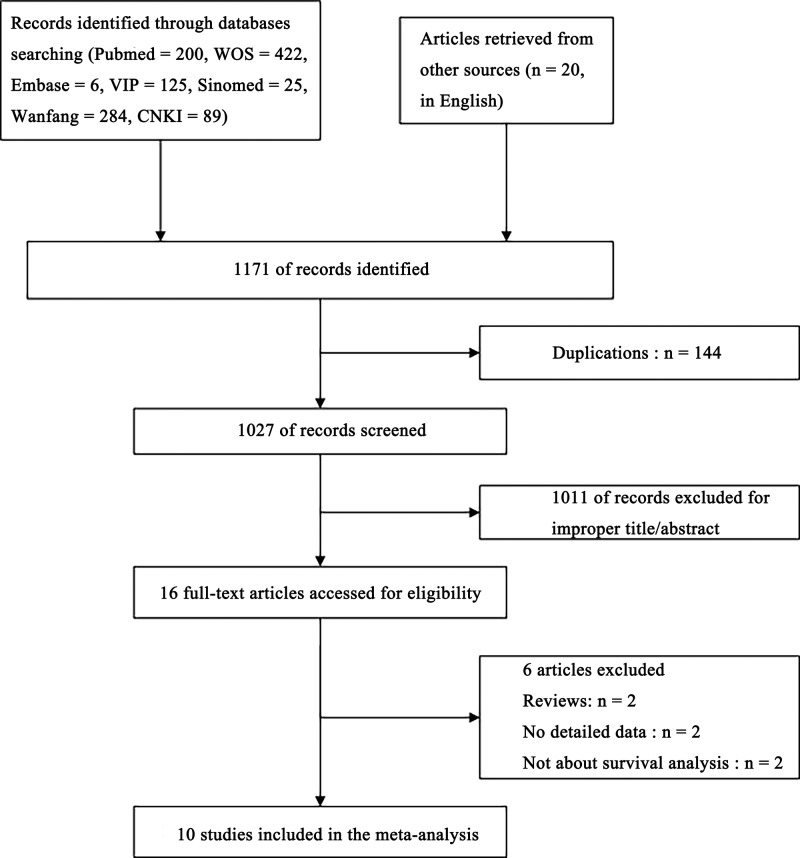
The flow diagram of the meta-analysis

By searching from electronic databases, references from reviews, articles, and other sources, we collected 1171 records in total (PubMed = 200, WOS = 422, Embase = 6 and VIP = 125, SinoMed = 25, Wanfang = 284 and CNKI = 89, other sources = 20) (Figure 1). First, 1155 articles were excluded after the title/abstracts were reviewed or duplications removing. Then, additional six articles were excluded after the full papers were evaluated. Finally, 10 articles were included in this meta-analysis.

The clinical features of the 10 included studies [12–20,32] are listed in Table 1. The articles were published between 2012 and 2018 with sample sizes ranging from 32 to 328 and included a total of 1510 participants. The majority of participants included in the studies were Chinese. Seven different types of cancer were examined (two of osteosarcoma, two studies of non-small cell lung cancer, two of renal cell carcinoma, one of hepatocellular carcinoma, one of esophageal squamous cell carcinoma, one of prostate cancer, and one of multiple myeloma). As for OS, DFS, PFS, and RFS, HRs and 95%CIs were directly retrieved from 6, 3, 1, and 1 studies, respectively, and were calculated from survival curves for 1, 0, 1, and 1 study, respectively. Eight studies measured the miR-19a expression level by qRT-PCR, while three studies applied the methods of miRNA array. OS, DFS, PFS, and RFS were extracted as survival outcome measures in 70% (7/10), 30% (3/10), 20% (2/10), and 20% (2/10) of the studies, respectively. The main characteristics of each study were summarized in Supplementary Additional file S1.

**Table 1:** Meta-analysis of miR-19a as a prognostic indicator for patients of various carcinoma

	No. of studies	No. of patients	Pooled HR(95%CI)		Meta regression	Heterogeneity	
			Fixed	Random	*P*-value	*I*^2^	*P*-value
Overall	7	1039	1.79(1.53, 2.09)	2.31(1.11, 4.83)		94.70%	0.000
Sample size					0.540		
<150	3	242	1.68(1.35, 2.08)	2.39(0.42, 13.57)		98.00%	0.000
≥150	4	797	1.92(1.53, 2.41)	2.16(1.22, 3.82)		94.70%	0.000
NOS scores					0.626		
<8	4	572	1.89(1.58, 2.27)	2.28(0.72, 7.21)		97.20%	0.000
≥8	3	467	1.53(1.13, 2.06)	2.20(0.99, 4.87)		69.00%	0.040
Specimen					0.535		
Tumor	5	642	1.73(1.42, 2.12)	2.52(0.77, 8.20)		96.10%	0.000
Blood	2	397	1.87(1.46, 2.40)	1.97(0.84, 4.58)		91.10%	0.000
Tumor category					0.073		
Osteosarcoma	2	198	7.17(5.04, 10.21)	7,17(5.04, 10.21)		0.00%	0.476
Non-small cell lung cancer	2	397	1.87(1.46, 2.40)	1.97(0.84, 4.58)		91.10%	0.001
Others	3	444	0.88(0.69, 1.13)	1.33(0.60, 2.92)		81.00%	0.005

*Abbreviations*: Fixed, fixed model; Random, random model.

### Association between miR-19a expression levels and overall survival

**Figure 2:**
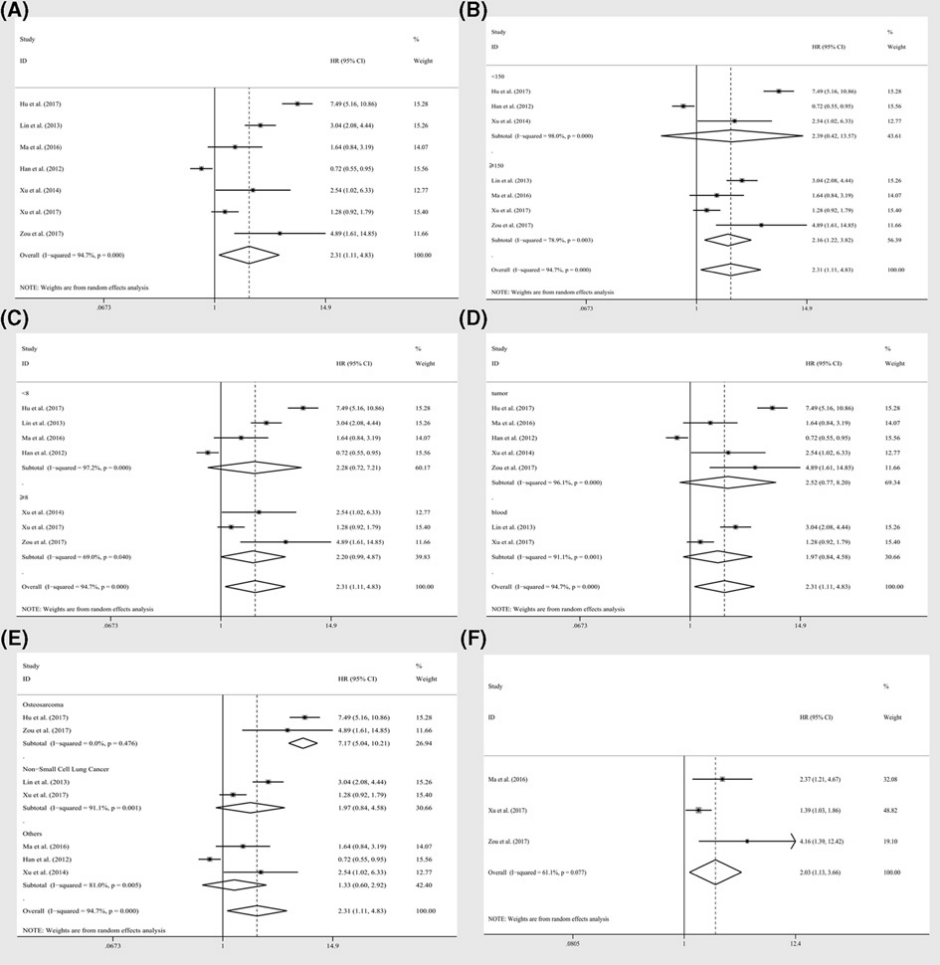
Prognostic effect of miR-19a expression levels on survival index The association between miR-19a expression levels and (**A**) overall survivals, subgroup analyses of (**B**) sample sizes (≥150 and <150), (**C**) NOS scores (≥8 and <8), (**D**) specimen (tumor or blood), (**E**) tumor category (osteosarcoma, non-small cell lung cancer, and others), and (**F**) DFS (data from log rank tests).

Seven enrolled studies including 1039 patients illustrated prognostic parameters (OS) with corresponding miR-19a expression levels. There was one study from Han et al. [32] that reported significant results in contradiction with other studies. In general, HRs of OS in fixed model revealed a significant association between miR-19a levels and OS (HRs = 1.79, CI: 1.53–2.09, Table 1), however there was a significant heterogeneity present within the studies (*I*^2^ = 94.70%, *P*<0.10, Figure 2A). Then the random pooling model was implemented and the significance of association between miR-19a levels and OS of cancer patients was consistent (HRs = 2.31, CI: 1.11–4.83, Figure 2A), suggesting the stableness of the results. In order to reduce the rather high heterogeneity, subgroup analyses were conducted by factors including sample size (≥150 and <150), NOS scores (≥8 and <8), specimen (tumor and blood), tumor category (osteosarcoma, non-small cell lung cancer, and others). As a result, the heterogeneity was controlled only in the osteosarcoma group (*I*^2^ = 0.00%, *P*=0.476, Figure 2E) and the relative significance was obvious (HRs = 7.17, CI: 5.04–10.21, Table 1). There were also significant associations between miR-19a expression levels and OS in the studies with sample size greater than or equal to 150 by random pooling model (HRs = 2.16, CI: 1.22–3.82, Figure 2B), while miR-19a seemed to be of no relation to OS in studies of different NOS scores and specimen by random pooling model (Figure 2C,D). In addition, significant associations were identified in studies with sample size less than 150 (HRs = 1.68, CI: 1.35–2.08), NOS scores less than 8 (HRs = 1.89, CI: 1.58–2.27) or greater than or equal to 8 (HRs = 1.53, CI: 1.13–2.06), specimen derived from tumor (HRs = 1.73, CI: 1.42–2.12) or blood (HR = 1.87, CI: 1.46–2.40) and the patients of non-small cell lung cancer (HRs = 1.87, CI: 1.46–2.40) by fixed pooling model (Table 1). But the heterogeneities within the subgroups were still significant, except for the osteosarcoma groups.

On top of this, meta regression of covariates analyses was further performed and a relatively significant contribution to the variation of HRs was recognized as the variate, which was tumor category (*P*=0.073, Table 1) (*P*=0.540 for sample size, *P*=0.626 for NOS score, *P*=0.535 for specimen, respectively). Moreover, the estimate of between-study variance tau-squared (*τ*^2^) decreased by 0.8840 to 0.2398 after the standardization of the variate (tumor category) in the overall analysis. However, the residual *I*^2^ for overall analysis was still significant, which was 80.27%.

**Figure 3:**
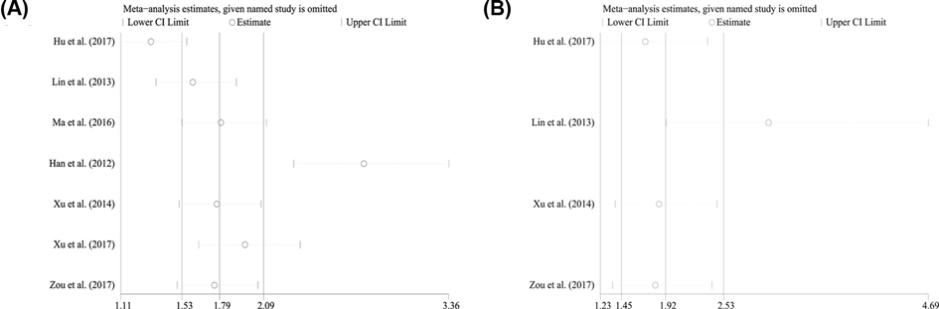
Sensitivity analyses for pooling results from univarite or multivarite statistics Sensitivity analyses for HRs of data for overall survivals extracted from (**A**) log tests and (**B**) cox multivariate regression.

Moreover, the sensitivity analysis was performed and no studies seemed to have great impacts on the significance of the results (Figure 3A). Since, the number of enrolled studies was less than 10, publication bias evaluation was not performed.

As shown in Table 1, two studies reported RFS, and another two studies revealed PFS, while three studies analyzed DFS including cox multivariate regression. Thus, after pooling the HR, we observed no significant association between miR-19a expression levels and RFS the random pooling model (HRs = 3.11, CI: 0.63–15.29, Table 2), PFS (HRs = 1.72, CI: 0.59–5.04, Table 2). However, significance was recognized of miR-19a expression levels in relation to DFS (HRs = 2.03, CI: 1.13–3.66, Figure 2F) with the data extracted from univariate analyses within corresponding studies, while the pooling results of data extracted from cox multivariate analyses revealed significance by fixed model (HR = 1.46, CI: 1.10–1.93, Table 2) instead of random model (HR = 2.00, CI: 0.98–4.09, Table 2). Compared with OS analyses, the heterogeneity among the studies was relatively lower but still significant (Table 2).

**Table 2:** Association between miR-19a expression levels and other prognostic indicators

	No. of studies	No. of patients	Pooled HR(95%CI)		Heterogeneity	
			Fixed	Random	*I*^2^	*P*-value
RFS	2	386	2.43(0.93, 6.33)	3.11(0.63, 15.29)	50.30%	0.156
PFS	2	190	1.48(0.80, 2.74)	1.72(0.59, 5.04)	60.50%	0.112
DFS						
Univariate	3	596	1.60(1.23, 2.08)	2.03(1.13, 3.66)	61.10%	0.077
Multivariate	3	596	1.46(1.10, 1.93)	2.00(0.98, 4.09)	69.70%	0.037

*Abbreviations*: Fixed, fixed model; Random, random model.

### The independent role of miR-19a expression level as prognostic indicator

**Figure 4:**
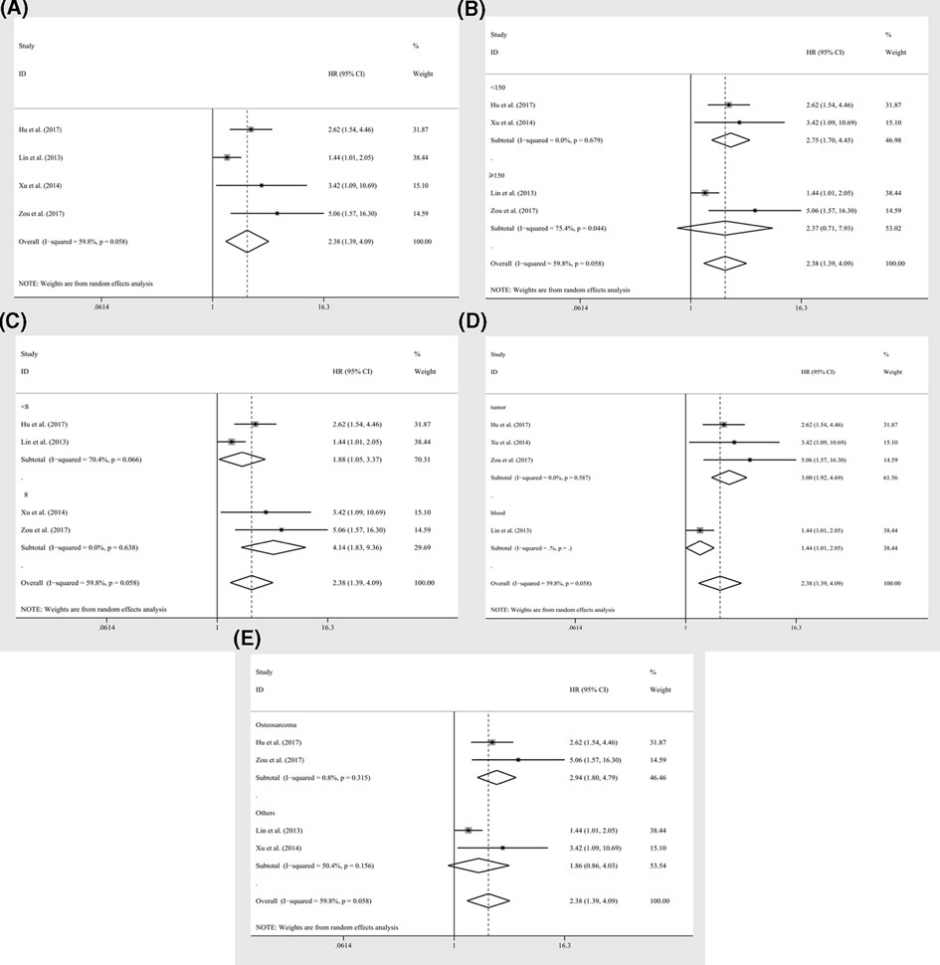
Independent prognostic efficacy of miR-19a expression levels on survival index The independent role of miR-19a as a prognostic detector for the overall survivals in patients of carcinoma, (**A**) overall survivals, subgroup analyses of (**B**) sample sizes (≥150 and <150), (**C**) NOS scores (≥8 and <8), (**D**) specimen (tumor and blood), (**E**) tumor category (osteosarcoma, non-small cell lung cancer, and others).

Four studies containing 504 patients applied cox multivariate regression to evaluate the independent prognostic role of miR-19a expression levels in cancer patients. With pooling strategy, the significant relation of miR-19a expression to the OS (HRs = 2.38, CI: 1.39–4.09, Figure 4A) was identified and the heterogeneity was relatively significant (*I*^2^ = 59.80%, *P*=0.058, Figure 4A). Similarly, to achieve homogeneity, subgroup analyses were applied and the heterogeneities were reduced within the studies of sample size less than 150 (*I*^2^ = 0.00%, *P*=0.679, Table 3), NOS scores greater than or equal to 8 (*I*^2^ = 0.00%, *P*=0.638, Table 3), specimen derived from tumor (*I*^2^ = 0.00%, *P*=0.587, Table 3), and the patients of osteosarcoma (*I*^2^ = 0.80%, *P*=0.315, Table 3). Also, the significant relations of miR-19a expression levels to OS were recognized within the studies of sample size less than 150 (HRs = 2.75, CI: 1.70–4.45, Figure 4B), NOS scores greater than or equal to 8 (HRs = 4.14, CI: 1.83–9.36, Figure 4C) or less than 8 (HRs = 1.88, CI: 1.05–3.37, Figure 4C), specimen derived from tumor (HRs = 3.00, CI: 1.92–4.69, Figure 4D) or blood (HRs = 1.44, CI: 1.01–2.05, Figure 4D), and the patients of osteosarcoma (HRs = 2.94, CI: 1.80–4.79, Figure 4E). Sensitivity analyses revealed that no studies had significant impacts on the results (Figure 3B). Due to insufficient studies, evaluation of publication bias was not performed.

**Table 3:** Meta-analysis of miR-19a as an independent prognostic indicator for patients of various carcinomas

	No. of studies	No. of patients	Pooled HR(95%CI)		Heterogeneity	
			Fixed	Random	*I*^2^	*P*-value
Overall	4	504	1.92(1.45, 2.53)	2.38(1.39, 4.09)	59.80%	0.058
Sample size						
<150	2	137	2.75(1.70, 4.45)	2.75(1.70, 4.45)	0.00%	0.679
≥150	2	367	1.60(1.14, 2.25)	2.37(0.71, 7.93)	75.40%	0.044
NOS scores						
<8	2	233	1.73(1.29, 2.33)	1.88(1.05, 3.37)	70.40%	0.066
≥8	2	271	4.14(1.83, 9.36)	4.14(1.83, 9.36)	0.00%	0.638
Specimen						
Tumor	3	303	3.00(1.92, 4.69)	3.00(1.92, 4.69)	0.00%	0.587
Blood	1	201	1.44(1.01, 2.05)	1.44(1.01, 2.05)	–	–
Tumor category						
Osteosarcoma	2	198	2.93(1.81, 4.76)	2.94(1.80, 4.79)	0.80%	0.315
Others	2	306	1.55(1.11, 2.18)	1.86(0.86, 4.03)	50.40%	0.156

*Abbreviations*: Fixed, fixed model; Random, random model.

### Correlations between miR-19a levels and clinicopathological features among various carcinomas

There are three articles containing 472 cancer patients investigated the association between miR-19a levels and various clinic characteristics. As shown in Table 4, lymph node metastasis was significantly associated with miR-19a expression levels (OR = 0.564, CI: 0.346–0.921). However, there were no significant relations observed of gender (OR = 0.998, CI: 0.655–1.521), age (OR = 0.962, CI: 0.566–1.637), smoking habit (OR = 1.270, CI: 0.759–2.124), and TNM stage (OR = 1.239, CI: 0.753–2.037) to the expression level of miR-19a. The homogeneity was achieved within all the groups. Sensitivity analysis and evaluation of publication bias were not available for insufficient number of recruited studies.

**Table 4:** Overall analysis of miR-19a expression association with clinicopathological characteristics

Clinicopathological parameters	No. of studies	No. of patients	Pooled OR(95%CI)		Heterogeneity	
			Fixed	Random	*I*^2^	*P*-value
Gender (male vs. female)	3	472	0.998(0.655, 1.521)	0.998(0.654, 1.522)	0.00%	0.761
Age (≥55 vs. <55 years)	2	271	0.962(0.566, 1.637)	0.962(0.566, 1.637)	0.00%	0.807
Smoking (never vs. ever)	2	306	1.270(0.759, 2.124)	1.270(0.760, 2.122)	0.00%	0.731
Lymph node metastasis (absent vs. present)	2	306	0.565(0.346, 0.921)	0.564(0.346, 0.920)	0.00%	0.422
TNM stage (III+IV vs. I+II)	2	306	1.239(0.753, 2.037)	1.239(0.753, 2.038)	0.00%	0.443

*Abbreviations*: Fixed, fixed model; Random, random model.

## Discussion

It is of great interest to explore prognostic biomarkers for patients with human malignancies as these biomarkers can help to directly stratify patients and effectively guide clinical decision-making. Recently, many studies have observed that miR-19a plays a key role in the progression and metastasis of human cancers [30,36–39]. Although Liu et al. [40] and Zhang et al. [41] have made a meta-analysis studying the relationship between miR-17-92 cluster (miR-17, miR-18a, miR-19a/b, miR-20a, and miR-92a) and human cancers, both of them only focused on the overall effects of all six mi-RNAs on cancer, instead of further analyzing the prognostic value of each mi-RNA by subgroup analyses, such as different specimens, sample sizes, cancer categories, etc. Thus, the conclusion provided by those studies was not detailed as expected. Also, the correlation between each miRNA expression and clinicopathological characteristics of cancer patients was not considered in both of the studies. Thus, the exact role of miR-19a on the clinical prognosis of patients in various human cancers still needs further recognition. To our knowledge, this is the most comprehensive meta-analysis that focuses on the prognostic value of miR-19a in various human malignancies.

Our results demonstrated that the significant association between miR-19a expression levels and OS was identified by fixed pooling model, however the heterogeneity was significant obvious. Then, we applied subgroup analysis to eliminate the heterogeneity form intrinsic variety such as sample sizes, NOS scores, and tumor category. As a result, the homogeneity was achieved within the osteosarcoma group, while the heterogeneity still exited within other subgroups. Moreover, the sensitivity analysis revealed that no studies could fluctuate the significance of the overall result, which indicated the stableness of the result. To further explore the source of heterogeneity, meta regression was implemented to identify significance of the between-study heterogeneity. Tumor category was recognized to generate the most significant impacts (*P*=0.073) on the pooling effects, which indicated that the source of between-study heterogeneity might have come from the variety of tumor types included in the study. After normalization of tumor category, the indicator of between-study, *τ*^2^, reduced from 0.8840 to 0.2398, and the diversity of tumor types included could explain the between-study variance by 61.37%. Thus, it suggested that the diversity of enrolled tumor types might be the major source of between-study heterogeneity. There might be more underlying variates that had aroused the heterogeneity among studies such as the follow-up time of survival analyses, the detection methods of miR-19a, the distribution of miR-19a expression levels among cancer patients, the residences and characteristics of the patients, and so on. However, we are unable to evaluate the bias caused by those factors for either lack of detailed information or difficulty of quantification. Moreover, the residual *I*^2^ was still significant (80.27%), which indicated that the heterogeneity within each study was still vital and could not be controlled by the meta-analysis. Also, we detected other markers as RFS, PFS, and DFS. Only the pooling data extracted from univariate analysis for DFS revealed significance, and the heterogeneities were also obvious. However, for lack of relevant data, we could not perform subgroup analyses, meta regression, sensitivity analyses, and so on to evaluate the source of heterogeneities. For the rather high heterogeneity, the pooling results from random model were relatively reliable. As a consequence, higher miR-19a expression level was significantly associated with poor clinic outcomes, especially for the patients of osteosarcoma.

The cox regression [42] has been proved to be effective in the survival analysis, because it evaluated the contribution of each factor by adjusting others, which suggested that the results indicated the independent role of each factors playing in the outcomes. However, the number of studies applying cox regression mode were relatively limited, compared with that of studies using log rank tests or Kaplan–Meier survival curves. As shown in Table 2, the independent prognostic role of miR-19a expression levels for cancer patients was significantly observed by both fixed and random pooling model, indicating the stableness of the result. But the heterogeneity was still significant. After utilizing subgroup analyses, the heterogeneities were successfully controlled for studies with sample size smaller than 150, NOS score greater than or equal to 8, specimen derived from tumor and patients of osteosarcoma. Sensitivity analysis was also performed and no studies had significant impacts on the results, which means the stableness and reliability of the results. Thus, over expression of miR-19a can serve as an independent indicator for poor prognosis and this result was consistent to the majority of the published articles reporting miR-19a as a promotor of cancer cells proliferation [43], tumorigenesis [44], migration [45], invasive [46] activity.

As a result, lymph node metastasis was found to be associated with miR-19a expression diversity, which suggested that cancer patient with over expressed miR-19a tended to develop lymph node metastasis. Nevertheless, no significance was identified of the association between miR-19a and several highlighted clinical features, such as gender, ages, smoking habit or TNM stages. MiR-19a expression levels were also found to be significantly related to venous or lymphatic invasion of esophageal squamous cell carcinoma [14], distant metastasis, anatomic location, and response to chemotherapy of osteosarcoma [16]. However, for lack of available studies, very limited amount of studies as well as features were recruited for the meta-analysis. More relevant researches were required to enrich the results and improve the reliability. Moreover, the analyses of clinic features of a specific cancer should be normalized for the cut-off values, the feature categories, and so on, so as to enrich the enrolled cases and characteristics for the meta-analysis.

There exist a few drawbacks that shall be clarified in our research. First of all, for lack of specific data in text for RFS, the relevant HRs and its confidence intervals of Fu et al. [17] extracted by the Kaplan–Meier Curves with Engauge Digitizer 9.8 as well as the spreadsheet calculator designed by Tierney et al. [33] did not support the significance claimed in the original article. The data were collected by two independent authors (Xiangyu Deng and Kaige Ma) from Fu et al. [17] for several times using the methods described above whose accuracy had been proved by many researches [47–49]. The extracted data were always consistent but significantly different from the original articles. The bias needed to be avoided by more precise data extracting methods or improving the quality of the enrolled studies. Second, the papers language was restricted to English and Chinese and may cause the bias due to lack of other populations. Third, the cut-off values of the expression levels of miR-19a were not precisely acknowledged among the studies, though most of them were presupposed as median. Fourth, the number of researches included was not sufficient enough. More relevant studies and patients should be identified for this analysis to enhance the reliability and confidence of our findings.

## Conclusions

Summarily, miR-19a expression levels could serve as a potential prognostic marker of the human cancer. The over expression of miR-19a was an underlying risk of poor prognosis in many human malignancies, especially in osteosarcoma. Moreover, elevated miR-19a expression was linked to the potential of lymph node metastasis. Due to relatively insufficient data, further studies in a larger scale of normalization are required to verify the findings.

## Availability of data and materials

The authors declare that all data supporting the findings of this study are available within this article and the enrolled articles for meta-analysis.

## Supporting information

**Additional file S1:** Characteristics of studies included in the meta-analysis.

## Abbreviations

CNKI: China National Knowledge Infrastructure
DFS: disease-free survival
HR: hazard ratio
HRs: pooled hazard ratios
miRNAs: microRNAs
NOS: Newcastle–Ottawa Scale
OS: overall survival
PFS: progression-free survival
qRT-PCR: real-time polymerase chain reaction
RFS: recurrence-free survival
*τ*^2^: tau-squared
WOS: Web of Science
95%CIs: 95% confidence intervals
